# Letter from Dr. A. C. Dayton, Dentist, of Vicksburg, Miss

**Published:** 1849-10

**Authors:** A. C. Dayton


### ARTICLE IV.

### *Letter from* Dr. A. C. Dayton, *Dentist, of Vicksburg, Miss.*

Editors of the Journal:

The following case of metastasis of the menstrual secretion from the uterus to the mouth, was related to me a few days since, by Dr. J. M. Jones, of Madison Parish, La. Thinking it worthy of note, I asked and obtained his permission to send it to you. If you think it worth publishing, please give it a place in the Journal.

It may, perhaps, be right to say, since I have no personal knowledge of the case enclosed, that I have no doubt of the veracity of Dr. Jones. The parties concerned live opposite this city, in Louisiana, and are all known here; and Dr. J. furnished me with references to persons in the neighborhood, of whom I might make further inquiries—this I did not consider necessary.

Yours, most respectfully,

A. C. DAYTON.

Case.—A negro woman, aged forty years, belonging to Alfred Johns, Esq., of Madison Parish, La., had, about fifteen years since, an attack of fever, soon after which her teeth became troublesome, and she had frequent spells of bleeding from the mouth. It was soon observed that these attacks returned at regular monthly intervals, and that the ordinary menstrual discharge had entirely ceased. Her general health became very bad, and she was unable to do any work. In the intervals of the bleeding, her face, abdomen and limbs had the appearance of one suffering from general dropsy. For a day or two before each return of the bleeding, she suffered greatly from pain in her head and face. The blood seemed to ooze out of the sockets around the roots of the teeth, and from the masses of fungous flesh which projected in several places from the swollen and spongy gums. The flooding at length became so profuse as greatly to debilitate her, and, indeed, to endanger her life at each return of it.

Dr. R. G. Parkam, a practicing physician of the parish, was called in, and, on learning the condition of her mouth, desired that Dr. Jones, a dentist, might be consulted. Dr. Jones found nearly all her remaining teeth decayed; some of them broken off and covered by the gums—the others loose in their sockets; the gums soft and spongy, and in several places shooting up in fungous masses, which bled freely from the slightest scratch. He at once removed eight of the teeth and roots, and after two weeks, five others, making thirteen in all. He then cut away the fungous projections from the gums, and directed the use of an astringent wash to the mouth, and the internal use of the muriated tincture of iron, twenty drops, three times a day. At the return of the next regular period, she had no bleeding from the mouth, and has not had any since. It is now about four months since the operation was performed. In three weeks she was able to take her place in the field, and has up to this time, enjoyed perfect health.

